# Capturing Change in a Community–University Partnership: The *¡Sí Se Puede!* Project

**Published:** 2005-03-15

**Authors:** Michele A Kelley, William Baldyga, Fabiola Barajas, Maria Rodriguez-Sanchez

**Affiliations:** University of Illinois at Chicago, School of Public Health, Division of Community Health Sciences; Associate Director, Institute for Health Research and Policy, Co-director, Illinois Prevention Research Center, University of Illinois at Chicago, Chicago, Ill; MacNeal Health Network, Berwyn, Ill; Illinois Prevention Research Center, University of Illinois at Chicago, Chicago, Ill

## Abstract

**Background:**

Community health interventions are increasingly employing partnerships combined with multilevel intervention models to achieve their objectives. Resources and methods for project evaluation are often limited to changes in population health status or health behaviors, while broader contextual questions that may illuminate mechanisms for change across ecological levels and project sustainability may not be addressed.

**Context:**

This paper describes a project to prevent and control diabetes in a Latino community and presents practical methods for addressing some challenges to evaluation, using data sources that often may be overlooked.

**Methods:**

A case study method was used to examine approaches to capture data that can help explain changes across ecological levels. An ecological framework was used to organize sources of data. Data sources and findings are related to project timelines and goals.

**Consequences:**

Although not a direct focus of the original research, substantial changes in community capacity were observed and measured over the course of the five-year project. Documentation on community change was found in routine project reports, logs, the news media, meeting minutes, and community documents.

**Interpretation:**

A logical progression of community change across ecological levels became evident. A modest post hoc evaluation was feasible, using data routinely available from project and target community sources. Specific questions for future research on how community change occurs and how such changes may relate to population health and sustainability are suggested.

## Background

Public health goals to reduce health disparities among people with chronic disease pose enormous challenges to health care providers, public health practitioners, academic researchers, and community leaders (1). In response to these challenges, recent community-based approaches to improve health are employing dual strategies of 1) creating partnerships between researchers and affected communities and 2) developing multilevel or ecological conceptual frameworks of health determinants. The rationale for the first strategy — creating community partnerships — is grounded in participatory democracy and recognition of local culture and community assets as necessary for tailoring acceptable and sustainable interventions (2). Although there is no agreed-upon definition of researcher–community partnerships (also referred to as community-based participatory research, or CBPR), key components include community involvement in the research process (i.e., identifying issues to be addressed) and in designing and delivering the interventions. In addition to improving the health behaviors of residents, CBPR aims to develop and strengthen community assets to address self-identified threats to health (3).

Although there is growing consensus that community engagement is a necessary and ethical condition for successful health promotion programs (4-6), there is a paucity of literature on the effectiveness of such engagement (7). The lack of literature may be explained in part by the complexity of and the insufficient detail on the partnerships described in research reports (8). Furthermore, creating partnerships is a necessary but insufficient strategy for improving health because many health determinants lie outside of the influence of the community (9).

Public health initiatives increasingly employ multilevel or ecological approaches to influence community-level change (10-12). McLeroy et al define ecological levels of influence on individual behavior (13). The ecological levels correspond to a series of community influences on intrapersonal factors (e.g., knowledge, attitudes, behavior, self-concept), interpersonal networks (e.g., family, friends, coworkers), institutional processes (e.g., formal and informal social networks, social support systems), community factors (e.g., relationships among organizations and networks), and public policy (e.g., local, state, and national laws and policies) (13). Although interest has grown in designing multilevel approaches to improve community health in areas such as nutrition, physical activity, and smoking prevention and cessation, no adequate, tested framework exists for measuring the effects of such initiatives beyond the individual or group level (14).

Sufficient knowledge of the dynamics and processes of change that occur in multilevel community health initiatives will provide future intervention efforts with an evidence base for making sound judgments on the best use of resources for addressing complex health issues. Because immigrant populations and communities of color are often targets for community-based interventions, multilevel frameworks could significantly enhance the ability of public health programs to close the gap on racial and ethnic health disparities.

In this paper, we demonstrate how a community-wide health promotion effort to prevent and control diabetes in a Latino community has addressed the challenges of capturing community change across ecological levels and the limitations in using available data. Like many chronic disease prevention research efforts, resources for evaluating this health promotion effort focused on measuring individual-level changes in health knowledge and behavior. (This evaluation is in progress.) Here we report on the use of existing data from project sources to perform a post hoc evaluation that captures observed differences in programs, participating organizations, and interorganizational relationships; we also document community-member participation in project development and leadership.

This paper reports on the initial project phase (1999–2004) and focuses on aspects of community change that have traditionally not been captured with data but may be critical in understanding conditions under which such projects are likely to influence individual health behaviors, to continue beyond the life of a specific project, and to enable the community to take on other related critical health issues as they emerge in the future.

We address the following questions: 1) What effects did the collaborative process have on the community? 2) How can we use data routinely collected during CBPR to describe community change beyond the individual level as the project evolves over time? We conclude with recommendations for data-capture strategies and suggest research questions to guide future community-based interventions that employ multilevel-change strategies.

## Context

This paper addresses the measurement of community change using a case study of a community-based diabetes prevention and control project. *¡Sí Se Puede!* (Yes We Can!, abbreviated as *SSP*) is a research demonstration project conducted by the Illinois Prevention Research Center (IPRC) at the University of Illinois at Chicago and funded through the Prevention Research Center Program Office and the Division of Diabetes Translation, Centers for Disease Control and Prevention (CDC). According to the CDC, "The Prevention Research Centers (PRCs) conduct participatory, community-based research to prevent disease and promote health. The outcomes are intended to be applicable to public health programs and policies" (15). The IPRC partnered with the Latino Organization of the Southwest (LOS) to conduct a community-based diabetes prevention and control project in two community areas on Chicago's southwest side. The LOS is a stable, engaged community organization dedicated to community improvement through empowerment strategies. The LOS provides leadership on a range of education, immigration, employment, housing, safety, social, and more recently, health issues.

The community of interest is known as Greater Lawn, an area of approximately 6.5 square miles in southwestern Chicago, Ill. Although a more detailed description of the community itself and the selection and engagement process are described elsewhere (16), it is important to note that the Latino community is only somewhat recently established on the southwest side of Chicago, as revealed by changes in the 1990 and 2000 censuses. This means that there were no prior organized community-wide health promotion efforts or mechanisms in place to foster such efforts.

In 1999, the IPRC and the LOS created the *SSP* Latino Diabetes Project with the goal of developing, implementing, and evaluating a program of activities designed to achieve the following: to raise awareness of the impact of diabetes on Latino patients and families, to enhance the ability of community members to reduce their risk of diabetes and diabetic complications, and to promote protective behaviors (healthy lifestyles) for Latino youth and their families. Four objectives guide the project activities toward this goal: 1) increase family and community awareness of the burden of diabetes and mitigating factors; 2) enhance behaviors that prevent diabetes onset or reduce diabetes complications; 3) improve the self-efficacy/self-management skills of diagnosed diabetics; and 4) enhance the quality of care delivered to diagnosed diabetics and increase opportunities to identify individuals at risk for diabetes. *SSP* interventions include health and diabetes education programs, school-based risk reduction curricula, physical activity programs such as walking clubs, nutrition education programs, health fairs, and a media campaign.

To address the objectives of the *SSP* project, a Community Advisory Board (CAB) was convened. The lead agency and project investigators nominated representatives of local schools and the local parks and recreation department, where project efforts were initially focused. The CAB is chaired by the director of the lead partner agency, LOS, and is composed of representatives of advocacy organizations, community nonprofit agencies, public schools, health care organizations, the faith community, local businesses, and resident program participants. Later, representatives from media organizations, local political leadership, and other organizations were added to ensure representation and assist with broad project objectives. A logic model that describes the inputs, outcomes, and impact of the *SSP* project is presented in the Figure.

**Figure:**
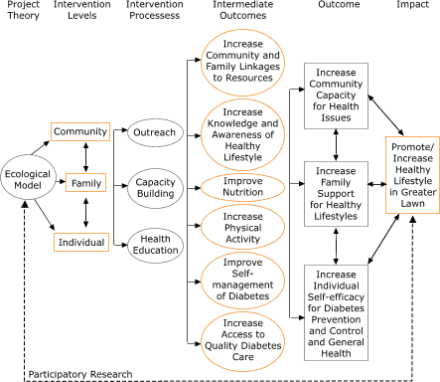
Logic model for the *¡Sí Se Puede!* project, Chicago, Ill.

IPRC researchers used assessment strategies to increase understanding of community leader and resident concerns. A series of focus groups with residents and in-depth, key-informant interviews with community leaders were conducted in 1999. Among the findings from interviews with community leaders was a consensus that bringing single-issue health expertise to the community, even on a high-priority topic such as diabetes, was not seen as sufficient for sustained community involvement. Related desires of community leadership included nurturing a value for health among community residents, integrating health concerns into the agenda of community organizations, and connecting community resources both within the target community and between the community and the broader metropolitan area. Subsequently, in 2001, a fifth objective was added: to develop the capacity of the community to address diabetes care and other self-identified health concerns. As initially conceptualized, enhanced community capacity was seen as a sum of the total effect of all intervention components rather than a separate research question, requiring its own design and data collection strategy.

IPRC researchers proposed analytic strategies to measure progress toward the first four objectives. Also proposed were educational objectives to strengthen community capacity by increasing knowledge of prevention research, for example, through attendance at national meetings. However, unlike other project objectives, no testable hypotheses or specific research questions were developed, and no analysis plan captured the activities related to community capacity or measured their impact. The primary reason was that resources for additional evaluation were not budgeted, as project resources were necessarily devoted to intervention activities and evaluation of the four original objectives. A secondary reason was related to the perception that "community building" would be opportunistic, responding to nonscientific (community) issues and could not be structured in a way that allowed for preplanned, quantitative measurement. Later, as the value of the activities conducted to increase community capacity became clarified and incorporated into project objectives, a strategy to catalogue and measure those changes was developed.

## Methods

Data capture strategies for this analysis (objective 5) relied on retrospective reviews of existing qualitative data collected routinely at CAB meetings and other meetings (meeting minutes) and through field notes, community leader interviews, and evaluation reports.

A community forum was held in the project's final months to assess how the *SSP* project influenced community organizations and their capacity to engage in diabetes health-promotion activities. An IPRC investigator led the discussion. Participants included community leaders representing various sectors, including education, health care, parks and recreation, and advocacy organizations. The discussion explored 1) reasons for joining a health-improvement initiative; 2) how the community thinks about health and diabetes; 3) how the community is organized for health promotion; 4) the relationships among organizations within and outside of the community; 5) the necessary skills and resources needed to improve community health; and 6) other issues of concern to the community.

Data from the forum and other sources were reported to the community through the CAB and presentations to other community advocacy organizations. Data about the program and community change were also found in local newspaper articles, television and radio programs, evaluation summaries from walking and nutrition and diabetes education programs, field reports, staff meeting notes, CAB meeting minutes, and focus group reports during program implementation. Although all of these activities were primarily concerned with how the program benefited individual residents, they also provided community-level contextual information that became the database for staff to begin to identify and catalog the reported community changes (beyond the individual level).

To ascertain the availability of information to describe community change, data from the project process and outcome evaluation were examined and categorized according to the corresponding ecological level. Table 1 provides a summary of the changes during the implementation of the *SSP* project. The table is organized according to selected ecological levels identified by McLeroy and colleagues (13); these levels are identified along the vertical axis: program, organizational, interorganizational, and community. The horizontal axis identifies the change observed, how it was observed, the source of data for the observation, the project objective addressed, and the time frame for the change.

## Consequences

This section reports *SSP* project results and data challenges related to increases in community capacity described at each ecological level. 

### Program level

For a health promotion program to be successfully established in a community, the program needs to be judged worthwhile, relevant to the intended audience, easily accessible, and reflective of the values and culture of the target audience. *SSP* program recruitment was built upon the recognition of the LOS as a credible organization within the community. *SSP* program activities were presented to community members as joint partnership activities with the LOS. To establish the *SSP* programs, the IPRC, with the approval and encouragement of community leadership, hired a staff member from a lead community-based organization to assist with program implementation and to voice community concerns. This led to increased organizational commitment for *SSP* and community resident acceptance of program initiatives. The *SSP* program also located an office at the LOS, creating a capacity previously unavailable in the community: dedicated staff and space within the community for health promotion activities.

As the *SSP* project progressed, several changes were observed at the program level. For example, walking club participants began to take on leadership roles. The program successfully encouraged participants to continue walking as a group after the initial eight-week walking program officially ended. In addition to becoming walking club leaders, *SSP* program participants have been active in promoting the *SSP* project, including joining the CAB.

Similarly, incorporation of community residents into the media campaign added to credibility and recognition of the project while creating successful role models for change. Community members volunteered to participate in the media campaign, telling of their own struggles to learn and maintain healthy behaviors and sharing their knowledge with other community residents in need of positive reinforcement.

In addition, program participants requested information on health topics not previously covered and suggested new or revised strategies, such as using existing English as a Second Language classes to deliver diabetes-related health education. This unique program configuration is an example of enhancing the *ecological validity* of an intervention: the program becomes part of the existing setting, rather than artificially imposed or interjected into a community (17,18). Because *SSP* programs are now integrated in familiar and trusted community structures, it is assumed they are more visible, accessible, and acceptable to the target population.

Perhaps the ultimate measure of enhanced program capacity is full institutionalization of a program within the target community. Community residents have become program leaders, replacing *SSP* staff to lead exercise programs. The transition from program participant to program leadership is an important change in community capacity and a necessary step to program institutionalization. Indigenous community leadership for program maintenance was not observed during the first three years of the project but became evident in the project's fourth year.

### Organizational level

Enhancing community organizational capacity is consistent with community leadership desires to play a lead role in health enhancement programs and with resident preferences for hearing messages from trusted community sources that speak to their experiences. Enhancing community organizational capacity creates change that reflects both an integration of critical categorical health issues into community organizations and the assumption of a leadership role in health for the primary community partner, the LOS. Project researchers believed that focusing on this community organization was the most effective way to enhance health capacity and sustainability.

Significant changes have occurred within the LOS as a result of engaging with the *SSP* project, including new emphasis on community health. The LOS has identified health as one of four major program areas, as reflected in its revised mission statement, and has created a health subcommittee to move forward its health agenda. LOS leadership and staff have participated in formal and informal training opportunities that have enhanced organizational capacity, and the LOS is now a focal point in the community for health promotion activities and information.

In addition to guiding the *SSP* project, the CAB continues to be an active forum for community health concerns. An ultimate goal for the *SSP* project will be to establish the CAB as an ongoing entity within the LOS, independent of *SSP* activities and funding.

Engagement with *SSP* has also fostered new health capacity within other community organizations. Local primary schools, a locus for *SSP* nutrition, activity, and educational programs, have increased awareness of student health needs and the relationship of good health to academic performance. Local school leadership is now a consistent and valued voice on the CAB, and schools participate in more health-promoting activities such as Walk Our Children to School Day, encouraging healthy eating, providing health education materials, and promoting community physical activity programs.

To a lesser extent, the local faith community, park district, and libraries have become more aware of health issues and the community's desire to address them. This increased knowledge has resulted in greater acceptance of health concerns as important to community residents and more willingness to collaborate on programs and activities.

Modification of the structure within each of the organizations identified above is important to helping sustain programs that support desired behavioral changes in individuals and families. The changes described became evident in years 3 to 5 of the *SSP* project, suggesting that community organizations required significant time to prepare, deliberate, and commit to engage in formal or informal health promotion activities.

### Interorganizational level

At the time the CAB was formed, the lead agency, the LOS, was part of a larger umbrella organization that focused on family and youth issues in the community. The *SSP* project focused on the LOS because the project's target audience was the rapidly growing Latino population in southwestern Chicago. The maturing of the LOS into a separate, nonprofit organizational entity gave the *SSP* project the necessary focus and organizational commitment required to move forward (16). At the interorganizational level, the *SSP* project has played a major role in developing significantly enhanced community capacity.

*SSP* activities to raise community awareness of the epidemic of diabetes and its differential impact on Latinos have resulted in 1) new relationships between the LOS and other community organizations, who are now collaborating on an increasing number of health-related issues and activities, including a community health fair; 2) new and expanded roles among existing partners, such as schools, libraries, and local businesses, which are now engaged in the dissemination of health-promotion information to community residents; 3) new linkages and access to resources previously unavailable or untapped, such as asthma education programs (asthma was identified in focus groups as a critical community concern), links to local health care provider organizations, and access to media outlets; and 4) creation of new networks of health-related projects and programs in the community. Referral patterns and resource networks have been expanded as organizations involved in *SSP* learn of each other's programs and attempt to coordinate and expand these resources through a community health coalition.

The *SSP* project has also resulted in changing perceptions about the community and its organizations. Through the community's involvement with *SSP*, health care providers and organizations, nongovernmental organizations, media outlets, and governmental organizations became aware of the community's interest in improving their health and identified the LOS as an agency with the knowledge, commitment, and resources to improve community health.

The *SSP* project provided the community a locus for its health-related concerns and the impetus and credibility to engage policy makers. LOS leadership has been recruited by nonprofit health organizations, local health care providers, and representatives of community organizations to participate in collaborative activities such as community forums and health education programs. The LOS has cultivated relationships among members of the news media, thus establishing access to news outlets and increasing opportunities for disseminating information. Community representatives and *SSP* staff members have been invited to join several local diabetes and Latino health-related coalitions. Our findings on the importance of community networks, coalitions, and linkages among resources in enhancing the effectiveness of health promotion initiatives are consistent with the literature (19,20). The interorganizational connections fostered through the *SSP* community–university partnership provide the community with a network of contacts to continue health improvement initiatives beyond the limited project period.

### Community level

For the *SSP* project, community-level changes reflect assessment and infrastructure development activities, including the development of the CAB. Changes at the community level were observed during the first year of the project. The community began to experience change by identifying health issues and recognizing diabetes as a concern. Prior to *SSP*, awareness of health as a community issue rather than an individual issue was not evident in community structures, nor did focus groups or key-leader surveys identify an organized response to any categorical health threats.

"Stages of change" is a concept that can be useful in describing a community's readiness to engage in health promotion (21). It parallels individual stages of change for health behavior as presented by Prochaska et al (22). Determining a community's stage of change can provide direction on strategies to guide and mobilize communities and advance through the next stages. In addressing community readiness issues, it is important to recognize that many community-based organizations address health within the context of social and economic conditions (e.g., immigration rights, housing, public safety) that affect the overall health of residents (23).

During its first year, the *SSP* community was at the "no awareness" stage regarding diabetes. By working with the community, *SSP* raised awareness of diabetes as a community issue. As the project matured, the community moved from the contemplation phase, or awareness of diabetes as a critical health issue, to the action phase when collaborative efforts to address health issues are evident, even if only at a beginning stage. The action phase is reflected in the development of a strategic plan by the CAB and the engagement of local political leadership to address diabetes as an important health concern. The development of a strategic plan did not occur until the fourth and fifth years of the project, suggesting that the community had to become knowledgeable about diabetes to effectively address concerns with local political leaders, including the city public health commissioner. All changes observed at the community level represent the initial steps toward building community capacity, and they are evident throughout Table 1.

### Interpretation

This paper describes a case study of observed changes in community capacity as a result of engagement in a community-based research program targeting lifestyle interventions for the prevention and control of diabetes in a Latino population. We attempt to address two issues: the effects of the partnership on the community as a whole, beyond the individual level, and how post hoc evaluation methods can capture aspects of change in community capacity.

A seminal paper by Goodman et al (24) outlines several dimensions of community capacity particularly relevant to our observations, including participation, leadership development, skills in intervention design and media advocacy, interorganizational networks and resource sharing, consensus building about values related to health, and organizational self-scrutiny related to strengths and limitations of health initiatives.

Although the importance of community capacity and its relationship to successful health initiatives has been established in the public health literature, measurement strategies and evaluation resources may not be available to many collaborative projects. Our evaluation of community capacity building, although not planned at the beginning of the *SSP* project, reveals modifications at the community level congruent with Goodman's dimensions. We therefore suggest strategies for measuring change in community capacity using existing data, when resources are limited, or when project planning precludes the use of formative evaluation strategies to capture change beyond individual behavior, knowledge, and skills. Finally, the paper has attempted to organize the data so that the sequential nature of capacity-generating activities is evident and so that timelines are clarified. This information can be useful in planning the next phase of interventions or in developing new interventions with different populations.

Although future project evaluation will need to confirm this, we believe that the momentum developed in the *SSP* project during the initial phase will be crucial to the project's success in the future as it evolves under new funding initiatives. Knowledge of initial community capacity change can be measured against future changes and can be used to ask focused questions about conditions within and external to the project (e.g., demographic and economic changes) that enabled or constrained the community in its ability to take action on issues critical to the health of its residents.

Ideally, the best practice in evaluating collaborative community-level interventions is to incorporate an evaluation plan at the beginning of the project that recognizes that community-level changes are distinct from individual-level changes and that collaborative processes are distinct from "intervention technologies," or activities designed to change knowledge and skills of individual participants (e.g., walking clubs). In many evaluation reports, it is difficult to distinguish between these separate components and outcomes, in part because they are not well defined or incorporated into logic models. A more specific conceptualization would allow for more focused questions and hypotheses so that researchers can share lessons learned in their attempts to capture change and relate change to program outcomes, both proximal and distal.

Research reports often lack details of collaborative efforts. Also underreported is information on broad community impacts and the consequences of how interventions were implemented. Trickett (8) underscores the importance of sufficient detail if we are to advance the science of collaborative, community-based research. We are challenged to document and "make what actually happens a heuristic for theory." Qualitative methods provide important tools to describe the process of change and the nature of change and to include perceptions and definitions of change from the vantage point of the community.

Ethnography particularly lends itself to participatory methods in that community members can be trained to work with researchers to document processes and issues during a project's evolution (25). Community member involvement is an intervention in and of itself, and the impact on the community workers needs to be documented (26).

In the next phase of scientific inquiry, hypotheses will need to be created to test proposed or alternative methods to enhance a community's ability to adopt and maintain healthier lifestyles. These methods can complement the quantitative strategies; each community intervention will have unique features that cannot be predetermined, controlled for (as in a randomized design strategy), or captured with a set of quantitative measures.

Another critical issue is a commitment of resources that adequately support collaborative work and that capture the processes and outcomes associated with it. Community-based collaborative research has a dual purpose of 1) empowerment and community development, also referred to as capacity building, and 2) the prevention and control of disease and improvement of health status (27-29). Labonte and Laverick (30) describe community capacity building in terms of its utility for more efficient delivery of interventions versus the "strengthened community action" that ensues as a desirable end in its own right. The inherent conflicts in this dual approach in addition to its methodological challenges and community benefits are becoming more apparent as evaluation strategies become more sophisticated in capturing total efforts (inputs and processes) and outcomes.

Ultimately, evaluation resources must be committed to long-term follow-up to capture these more distal effects. With these challenges in mind, we conclude with a proposed set of questions that may be useful in thinking through evaluation strategies at the beginning of a project: 

- What does the community desire for the future health of its residents and how has this changed over time?
- What are the characteristics of community roles and relationships in this project? How do these characteristics change over time as the project is implemented (e.g., intensity, duration)?
- How has community involvement (e.g., individual participants, organizations and coalitions, media) with the project changed over time and what accounts for this change?
- What are the effects of collaboration on the "intervention technologies" that were introduced to this project?
- What are the apparent effects of collaboration on community organizations, organization structures and mission, programs, coalitions, leadership, skills, and readiness to engage in health promotion efforts? 
- What community structures and resources were most useful in achieving project objectives for individual level changes?
- What unanticipated community changes occurred (both desirable and undesirable) during the project and what evidence is there that they are attributable to the project?
- What are community perceptions of the project and its accomplishments and how have these perceptions changed over time as the project was implemented?
- What aspects of community change appear to be most important for sustainability of the project and its constituent activities?

And finally:

- To what extent can community-level changes in capacity be transferred from one categorical health issue to future, emerging health issues?

## Figures and Tables

**Table:** Ecological-Level Community Changes Observed During the SSP Project Implementation^a^

**Ecological Level**	**Nature of Change**	**How Observed**	**Sources of Data**	**Project Objective**	**Time Frame**
**Program**	*SSP* project hires staff member from lead CBO	Acceptance of position	Appointment papers	e	Year 1
	*SSP* has joint offices at CBO and university	*SSP* staff have dual roles and presence at university and CBO; informational telephone number established at community organization, publicized in community	Staff schedule	e	Year 2-5
	Existing CBO programs (ESL) are used to deliver health information	Focus groups; community forum	Focus groups report; forum report	a, e, b	Year 4
	Community members take expanded roles in programs/activities (e.g., CAB, walking clubs)	Community participants volunteer to lead/participate in activities	Field notes, staff meeting minutes	a, e, b	Year 4
**Organizational**	Lead CBO establishes a health subcommittee	Included in their mission statement	Mission statement	a, e	Year 3
	Schools increase awareness of health concerns	Organizations seek additional health expertise; establish and participate in Walk Our Children to School Day; school offers additional physical activity schedule for students	Field reports; program evaluation data	a, b, e	Year 3-5
	Religious institutions collaborate and host programs	Organizations participate in additional health programs	Field reports; program evaluation data	a, b, e	Year 3-5
	Park district hosts yearly health education event	Attendee registration	Registration forms; newspaper articles	a-e	Year 3-5
	Libraries support distribution of health information at their agency	Project ideas are discussed and developed with library	Log of health education materials	a, b, e	Year 1
	School establish an onsite resource center for parents	Project ideas are discussed and developed with schools	Field reports; program evaluation data	a, b, e	Year 4-5
**Inter-** **organizational**	Director of CBO becomes consultant with School of Public Health Prevention Program	Personal communication with CBO	Director of CBO	e	Year 5
	Project leadership shifts from a community partnership to single agency (Latino-serving CBO)	Strong support and commitment for project from Latino-serving CBO	Letters of support; collaboration on various activities; meeting minutes	a, c, e	Year 2
	CBO develops health media links	Articles published in press; PSAs; radio spots; and live remotes	Local television, magazine, newspaper articles	a, c, e	Year 2-5
	CBO joins national/regional/local health organizations	Appointments to committees, work groups, coalitions	Meeting minutes (staff, CAB)	a, e	Year 1-5
	CBO develops new/enhanced relationships with local providers	Meetings with local providers; development of resource directory	CAB meeting minutes; community resource guide	d	Year 1-5
	CAB membership represents business, health, education, religious, NGO sectors	Participants discuss formation of local health coalition; community forum	CAB meeting minutes/focus group notes; forum report	e	Year 2-5
**Community**	Identified priority health concerns with the community	Focus groups, community leader interviews, and survey	Focus group reports, community leader interview reports, survey report	a, b	Year 1-2
	Increase political involvement in health issues	Representative from Alderman’s office on board; meetings with public health commissioner, local political leaders; community forum	Observation; forum report	a, e	Year 3-4
	Establish health community advisory board (CAB)	Monthly meetings, agendas	CAB meeting minutes	a, e	Year 1-5
	Residents use local health clubs, park district, CBOs for physical activity	Focus group	Focus group report; field notes	b	Year 3-5
	Developed strategic plan for CAB	Monthly meetings, agendas	CAB meeting minutes	a, e	Year 4-5

a CBO indicates community-based organization and here refers to the lead community partner (Latino-serving) organization; ESL indicates English as a second language; CAB indicates community advisory board; NGO indicates nongovernmental organization; PSAs indicate public service announcements. Program objectives are indicated by the following: Increase family and community awareness of the burden of diabetes and mitigating factors. Enhance behaviors that prevent diabetes onset or reduce diabetes complications. Improve the self-efficacy/self-management skills of diagnosed diabetics. Enhance the quality of care delivered to diagnosed diabetics and the opportunities to identify individuals at risk for diabetes. Develop the capacity of the community to address diabetes care and other self-identified health concerns.
